# Fabrication of Superhydrophobic and Luminescent Rare Earth/Polymer complex Films

**DOI:** 10.1038/srep24682

**Published:** 2016-04-18

**Authors:** Zefeng Wang, Weiwei Ye, Xinran Luo, Zhonggang Wang

**Affiliations:** 1 Department of Polymer Science and Materials, School of Chemical Engineering, Dalian University of Technology, Dalian 116024, China

## Abstract

The motivation of this work is to create luminescent rare earth/polymer films with outstanding water-resistance and superhydrophobicity. Specifically, the emulsion polymerization of styrene leads to core particles. Then core-shell-structured polymer nanoparticles are synthesized by copolymerization of styrene and acrylic acid on the core surface. The coordination reaction between carboxylic groups and rare earth ions (Eu^3+^ and Tb^3+^) generates uniform spherical rare earth/polymer nanoparticles, which are subsequently complexed with PTFE microparticles to obtain micro-/nano-scaled PTFE/rare earth films with hierarchical rough morphology. The films exhibit large water contact angle up to 161° and sliding angle of about 6°, and can emit strong red and green fluorescence under UV excitation. More surprisingly, it is found that the films maintain high fluorescence intensity after submersed in water and even in aqueous salt solution for two days because of the excellent water repellent ability of surfaces.

Artificial superhydrophobic materials with water contact angles over 150° and water droplets easily rolling off the solid surface^1^,^2^,^3^,^4^,^5^,^6^,^7^ has attracted growing interest since it brings about unprecedented properties to inorganic and organic polymer materials such as self-cleaning^8^,^9^, anti-icing^10^ and anti-bacterial^11^, etc. Over the past decade, numerous superhydrophobic materials have been reported^12^,^13^,^14^,^15^,^16^,^17^,^18^,^19^,^20^, but there are still increasing demands for novel function to further extend their practical applications. For example, rare earth complexes have been widely employed in optical displays, fluorescent probe, luminescent labels and medical diagnosis fields^21^,^22^,^23^,^24^,^25^,^26^,^27^,^28^,^29^. Nevertheless, their fluorescence stability is always a great concern because rare earth complexes are intrinsically hydrophilic and the fluorescence property is liable to deteriorate in the moist or aqueous environments due to the ligand substitution by water molecules^30^,^31^,^32^. In this aspect, superhydrophobic modification, i.e., making the rare earth materials non-wettable, may provide a feasible solution to the above problem. For a superhydrophobic material with Cassie state, the large amounts of air bubbles existing on the interface between water and surface can prevent water from touching the materials, and thus effectively enhance the resistance to water corrosion.

Low-surface-energy substance and proper roughness are two essential factors for constructing superhydrophobic surfaces. Fluorine-containing polymers such as polytetrafluoroethylene (PTFE) are ideal candidates because they are commercially available and have large water contact angles over 110° as well as many other merits like low toxicity, high temperature stability and chemical resistance^33^. On the other hand, the previous studies have revealed that the hierarchical structure with multi-scale roughness in the range from micrometer to nanometer is advantageous for achieving a superhydrophobic surface with water contact angles over 150° and stable Cassie-Baxter state^7^,^34^,^35^,^36^.

With the above considerations in mind, the present work was undertaken to create rare earth/polymer films with fascinating superhydrophobic, water-resistant and luminescent properties. To this end, at first, spherical polymer nanoparticles with carboxylic groups covalently bonded in the outer layer were synthesized. The carboxylic groups provide sites to coordinate with various rare earth ions to produce core-shell-structured rare earth polymeric nanospheres (ca. 100 nm), which were then utilized to complex with PTFE microparticles (ca. 5 μm) in ethanol to obtain latexes. The latexes were cast on slide glasses to prepare superhydrophobic and luminescent polymer films. The focus of this work is to (i) synthesize and characterize the rare earth-coordinated polymer nanoparticles; (ii) study the microstructure of micro-/nano-sized PTFE/rare earth polymer complex films; (iii) study the superhydrophobic and self-cleaning properties of the films; (iii) study the luminescent property and stability of films in water and aqueous salt solution.

## Results

### Synthesis and characterization of core-shell rare earth polymer nanoparticles

**Figure 1:**
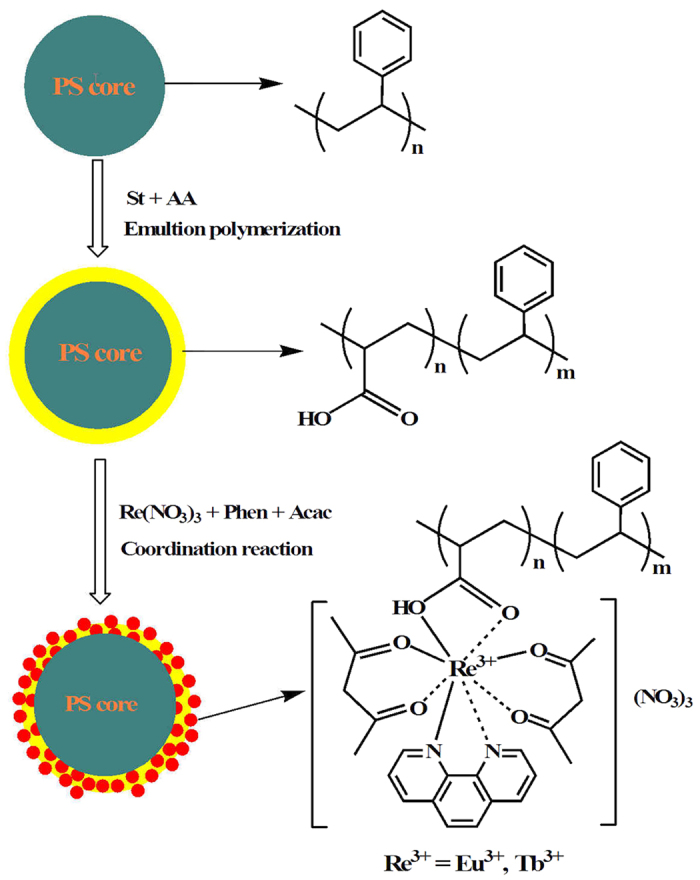
Chemical structures and synthesis routes to core polymer particle, carboxyl-containing core-shell polymer particle, and rare earth-coordinated polymer particle.

The synthesis of rare earth-coordinated spherical polymer nanoparticles are mainly composed of two steps as depicted in Fig. 1. The first step involves the preparation of core-shell-structured polymer nanospheres using polystyrene (PS) as core and polystyrene-co-poly(acrylic acid) (PS-*co*-PAA) as shell through emulsion step-polymerization technique. In the second step, the resultant nanoparticles with carboxylic groups in the shell layer were coordinated with Eu^3+^ and Tb^3+^ ions to obtain rare earth-coordinated polymer nanospheres denoted as Nano-Eu^3+^ and Nano-Tb^3+^, respectively. 1,10-Phenanthroline (Phen) and acetylacetone (Acac) were used as co-ligands to meet the coordination number of rare earth ion. Moreover, Phen and Acac are well-known light-harvesting compounds and have strong energy transfer ability to rare earth ions, which are helpful to enhance luminescent intensity of the rare earth complexes^37^.

The chemical structures of the carboxyl-containing polymer particles before and after coordination with rare earth ions were characterized by FTIR spectroscopy and elemental analysis. As shown in Supplementary Figure S1, the absorption at 1600 cm^−1^ is attributed to the benzene rings in PS segments, whereas the band at 1701 cm^−1^ belongs to the carboxylic groups in PAA moiety. After coordination reaction, for Nano-Eu^3+^ as an example, the absorption at 1701 cm^−1^ disappears and a new peak at 1380 cm^−1^ emerges due to the occurrence of coordination between Eu^3+^ and carboxylic group^38^. In addition, the comparison of FTIR spectra of Nano-Eu^3+^, Phen and Acac reveals that the stretching vibration of carbonyl in Acac at 1728 cm^−1^ red-shifts to 1519 cm^−1^, while the bent vibration of C-N bond in Phen at 1415 cm^−1^ and the skeleton vibration at 856 cm^−1^ red-shift to 1321 cm^−1^ and 845 cm^−1^, respectively, demonstrating the formation of coordination bonds of Eu^3+^ with nitrogen atom of Phen and oxygen atom of Acac^39^,^40^,^41^. The elemental compositions of the rare earth polymer particles were examined by elemental analyzer and inductively coupled plasma methods. The data in Supplementary Table S1 show that the measured values of C, H, N and rare earth ions agree well with the calculated values.

**Figure 2:**
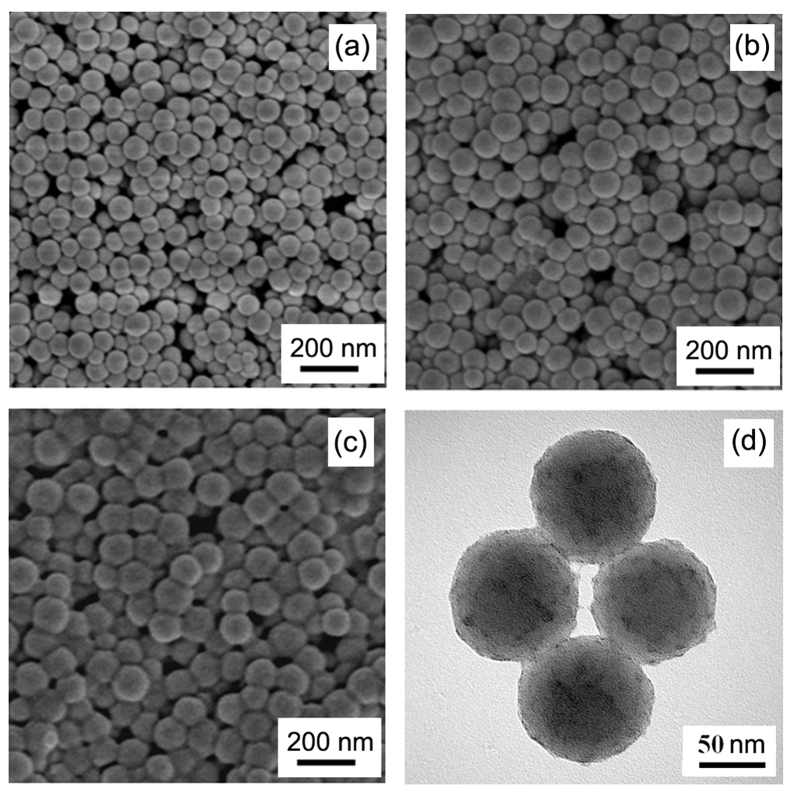
FE-SEM images for core polymer particles (**a**), carboxyl-containing core-shell polymer particles (**b**), rare earth-coordinated polymer particles (**c**), and HR-TEM image for rare earth-coordinated polymer particles (**d**).

The sizes and distributions of the resultant various particles were studied by dynamic light scattering (DLS), field-emission scanning electron micrograph (FE-SEM) and high-resolution transmission electron micrograph (HR-TEM). As seen in Supplementary Figure S2, relative to the core particle, the DLS curve of core-shell polymer particles obviously shifts toward right, suggesting that PS-*co*-PAA layer has indeed grown on the surface of PS core. Moreover, after coordination, a new rare earth-coordinated polymer layer has covered outside the particle, leading to the further increased size. The z-average diameters of core, core-shell polymer particle and rare earth-coordinated particles are 83, 111 and 127 nm with the polydispersity indices of 0.090, 0.091 and 0.089, respectively, displaying quite narrow monomodel distribution. The FE-SEM images (Fig. 2a–c) show that the sizes of cores, core-shell particles and rare earth-coordinated particles also increase sequentially. In addition, the rare-earth nanoparticles are spherical and uniform as observed in HR-TEM images (Fig. 2d), which indicates that the rare earth moieties (black color) are on the surface of polymeric sphere.

### Surface morphology and thermal stability of PTFE/rare earth films

The polymer colloidal solutions consisting of PTFE microparticles and Nano-Eu^3+^ nanoparticles were casted on slide glasses to obtain PTFE/rare earth polymer complex films (PTFE-Eu^3+^), in which the weight ratios of PTFE to Nano-Eu^3+^ varied in a wide range from 0.1:1 to 20:1. The PTFE-Tb^3+^ films were also prepared in the same method. The microstructure including surface morphology and chemical composition as well as thermal stability for the representative sample with weight ratio of PTFE to Nano-Eu^3+^ of 5:1 were studied by energy-dispersive X-ray spectroscopy (EDX), FE-SEM and thermal gravimetric analysis (TGA).

**Figure 3:**
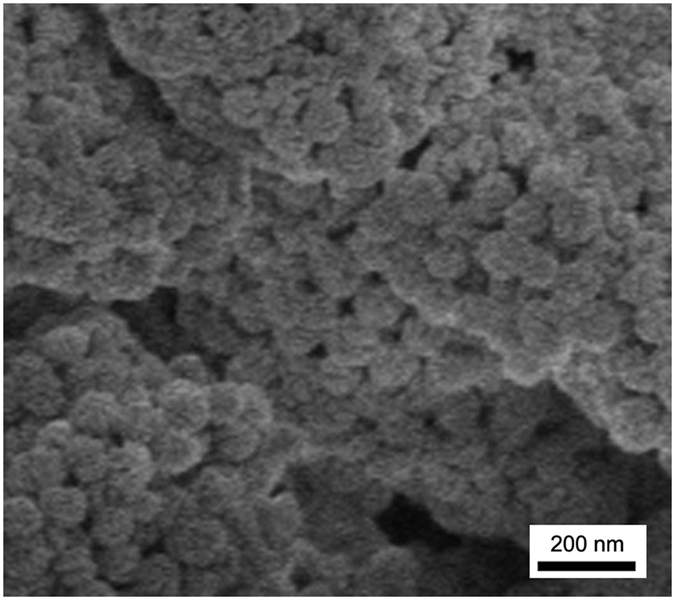
FE-SEM image of PTFE-Tb^3+^ film with the weight ratios of PTFE to Nano-Eu^3+^ of 5:1.

**Figure 4:**
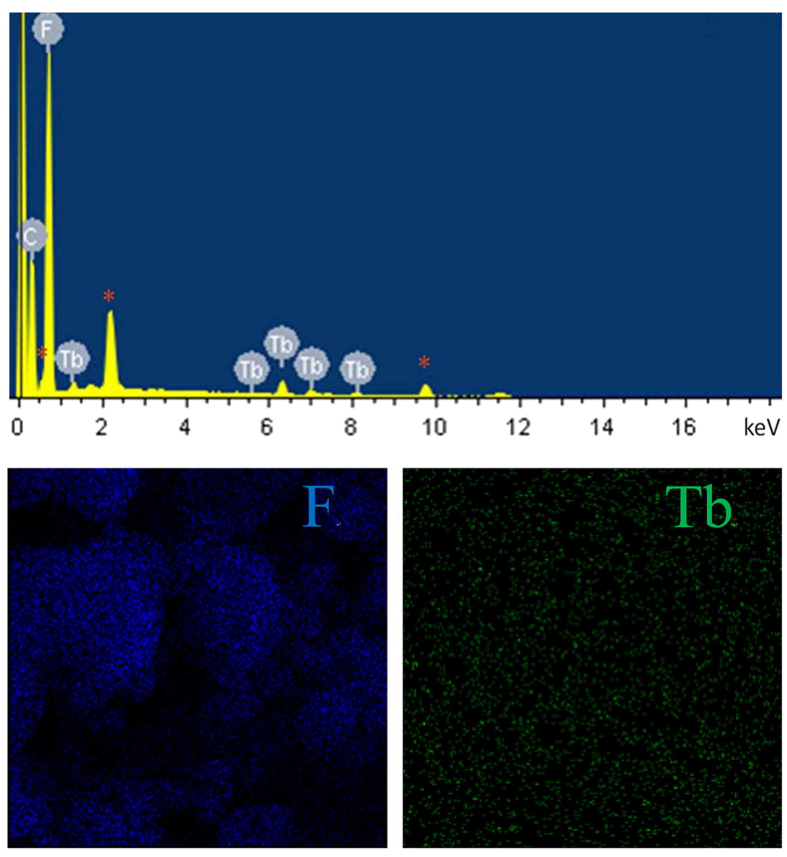
EDX spectrum (top) and mapping images (bottom) for PTFE-Tb^3+^ film.

As shown in Fig. 3, the PTFE-Tb^3+^ film exhibits a hierarchical mciro-/nano-sized rough morphology composed of PTFE microparticles and Nano-Tb^3+^ nanoparticles. Figure 4 displays that the F and Tb elements homogeneously distribute on the film surface. 

The thermal properties of PTFE-Eu^3+^ film and the neat Nano-Eu^3+^ film were evaluated by TGA in N_2_ atmosphere (Supplementary Figure S3). For Nano-Eu^3+^ film, the temperatures corresponding to the initial decomposition (T_i_) and maximum degradation rate (T_max_) are 166 and 417 °C, respectively. In contrast, PTFE-Eu^3+^ shows greatly increased T_i_ (296 °C) and T_max_ (564 °C). The reason is attributed to that PTFE particles play a shielding role in retarding heat transfer across the composite film, leading to an improved thermal stability. Besides, it is noted that the residual weight of Nano-Eu^3+^ is apparently higher than that of PTFE-Eu^3+^ because of the higher content of inorganic component in Nano-Eu^3+^.

### Superhydrophobic and self-cleaning properties of PTFE/rare earth films

**Figure 5:**
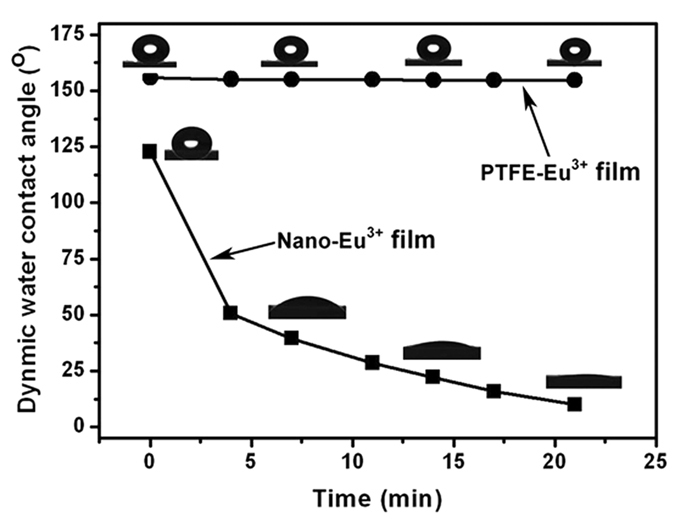
Variation of water contact angles with measuring time for the neat Nano-Eu^3+^ film and the PFFE-Eu^3+^ film with weight ratio of PTFE to Nano-Eu^3+^ of 5:1.

The wettabilities of rare earth polymer films with and without adding PTFE microparticles were studied by dynamically observing the variation of water contact angles with time (Fig. 5). For the neat Nano-Eu^3+^ film, the initial water contact angle (θ) is 123 ± 1.8° but the water droplet is not stable. The θ values rapidly decrease with time and the final value is 10 ± 2.6° after 21 min. The reason is that the rare earth-coordinated Nano-Eu^3+^ film is hydrophilic so that the capillary effect due to the packing of spherical Nano-Eu^3+^ particles induces the water droplet to seep from the film surface into the interior, resulting in the extremely low contact angle^42^. However, the hydrophobicity of films is significantly improved after incorporating PTFE microparticles. For the sample with the weight ratio of PTFE to Nano -Eu^3+^ of 5:1, the PTFE-Eu^3+^ film exhibits a water contact angle of 156 ± 1.2°, and the θ values remain almost constant in the time interval of 21 min. The superhydrophobic characteristic of the complex film could be attributed to two reasons: first, the film constructed with PTFE microparticles and Nano-Eu^3+^ nanoparticles has a multi-scaled roughness as revealed by the FE-SEM; second, the low-surface-energy PTFE particles (18.5 mN/m)^43^ effectively inhibit the penetration of water into the interior of the film.

**Figure 6:**
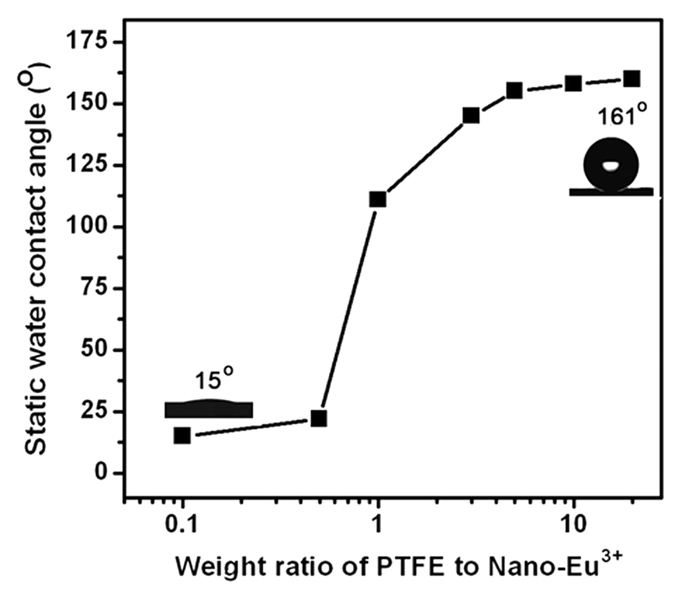
Dependency of static water contact angles for PFFE-Eu^3+^ films on the weight ratios of PTFE to Nano-Eu^3+^ for PFFE- Eu^3+^ film with the weight ratio of PTFE to Nano-Eu^3+^ of 5:1.

In fact, the hydrophobic property of the PTFE-Eu^3+^ films considerably relies on the PTFE content. As illustrated in Fig. 6, with the introduction of PTFE microparticles, at the initial stage, the contact angles slowly increases, but the θ values rapidly rise when the weight ratios of PTFE to Nano-Eu^3+^ surpass 1:1, and can reach 161 ± 1.6° when the ratio reaches 20:1.

**Figure 7:**
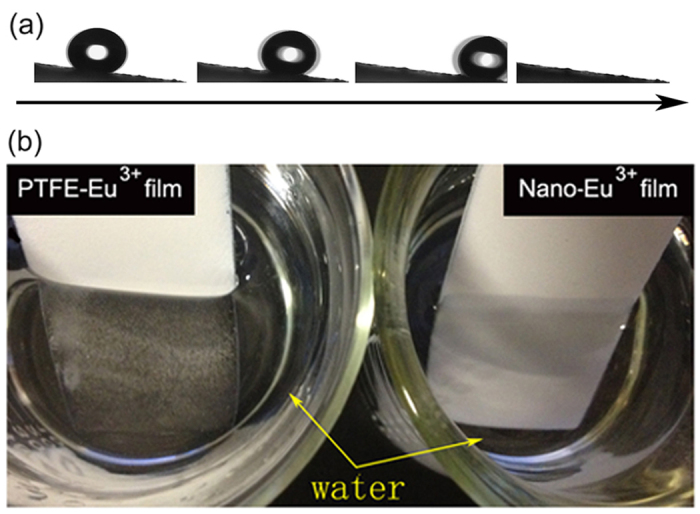
(**a**) Illustration of a water droplet rolling on the tilted surface at about 6°, and (**b**) images of PTFE-Eu^3+^ and Nano-Eu^3+^ films submersed in water.

On the other hand, for the sample with the weight ratio of PTFE to Nano-Eu^3+^ of 5:1, Fig. 7a shows that the water droplet easily rolls off the tilted PTFE-Eu^3+^ film with a angle of about 6°. It is rational to anticipate that the sliding angle can further decrease with the increase of PTFE content. The very low sliding angle indicates good self-cleaning property^44^. In addition, the film exhibits a mirror-like surface (Fig. 7b) when submersed in aqueous solution caused by the total internal reflection of light owing to the tiny air bubbles entrapped on the interface between the surface and water^45^,^46^. As a comparison, for Nano-Eu^3+^ film, the mirror-like appearance cannot be observed. It is seen that the PTFE-Eu^3+^ film is completely non-wettable, whereas the Nano-Eu^3+^ film is wet when pulled out of the water (Supplementary Figure S4).

### Fluorescence properties of the PTFE/rare earth films

**Figure 8:**
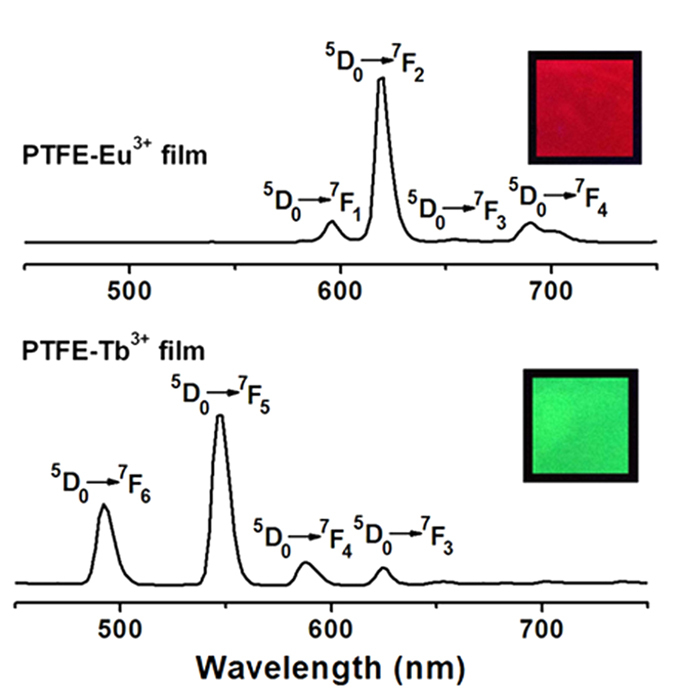
Fluorescence emission spectra of PTFE-Eu^3+^ and PTFE-Tb^3+^ films (insets are the luminescent images of films under the UV lamp at 365 nm).

Figure 8 exhibits the fluorescence emission spectra of PTFE-Tb^3+^ and PTFE-Eu^3+^ films under the excitation at 365 nm. The two films emit strong characteristic lights of rare earth ions (inset images). In the spectrum of PTFE-Eu^3+^, the peaks at 594, 618, 653 and 700 nm are assigned to ^5^D_0_ → ^7^F_1_, ^5^D_0_ → ^7^F_2_, ^5^D_0_ → ^7^F_3_ and ^5^D_0_ → ^7^F_4_ transitions of Eu^3+^ ion, respectively^21^. Among them, the intensity at 618 nm from the ^5^D_0_ → ^7^F_2_ transition is the strongest with a very narrow half-peak width less than 10 nm, suggesting good color purity. Similarly, PTFE-Tb^3+^ film emits characteristic green light, and the four peaks located at 491, 546, 586 and 622 nm are associated with the ^5^D_4_ → ^7^F_J_ (J = 6, 5, 4, 3) transitions of Tb^3+^ ions, respectively^21^.

**Figure 9:**
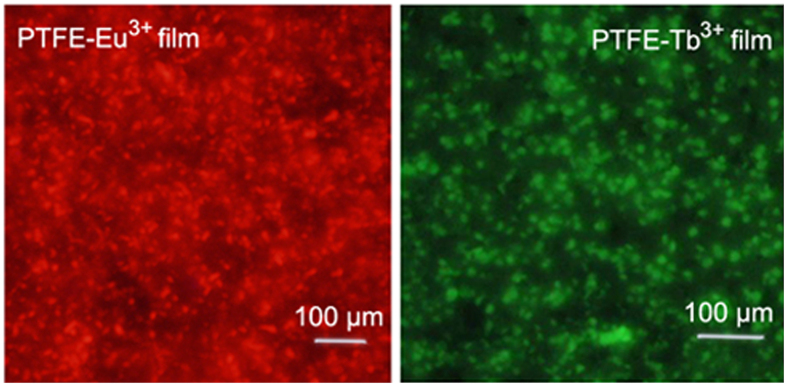
Images of fluorescence microscopy for PTFE-Eu^3+^ and PTFE-Tb^3+^ films.

The images of fluorescence microscopy show that PTFE microparticles covered with Nano-Eu^3+^ or Nano-Tb^3+^ particles are homogeneous in the films (Fig. 9). The PTFE particles in the films act as physical buffer to well separate the rare earth particles from each other to effectively avoid the occurrence of fluorescence quenching.

### Water-resistance of the PTFE/rare earth films

**Figure 10:**
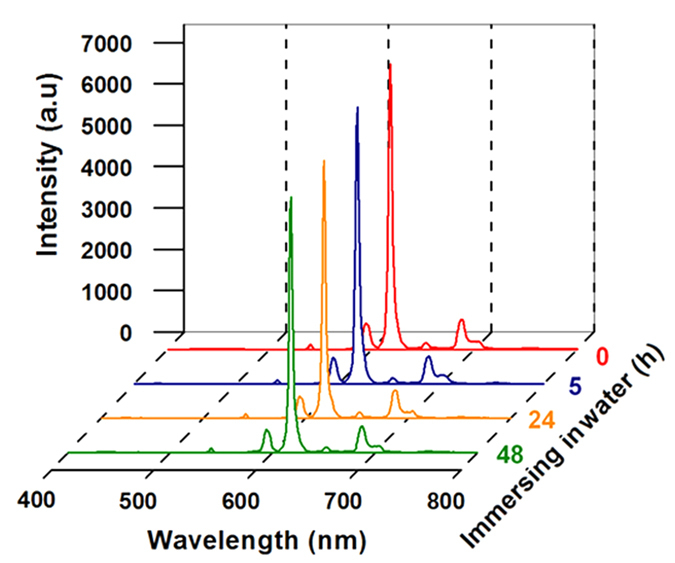
Fluorescence emission spectra of PTFE-Eu^3+^ films after submersed in water for different time.

The water-resistance and fluorescence stability of PTFE-Tb^3+^ films were evaluated by submersing the samples in water at room temperature for 5 h, 24 h and 48 h. Surprisingly, their fluorescence intensities almost unchange (Fig. 10). As a comparison, for the Nano-Eu^3+^ film without complexing with PTFE particles, its fluorescence intensity rapidly drops from 3725 a.u to 587 a.u, and the values continually decrease with the immersing time (Supplementary Figure S5). Furthermore, the similar experiments were conducted by submersing the samples in 20% aqueous NaCl solution. It is seen that the rare earth polymer films can also maintain high fluorescence stability (Supplementary Figure S6). The excellent stability of the PTFE/rare earth polymer film in aqueous environment is believed to be tightly related to its Cassie-stated superhydrophobic surface. The water-repellent surface effectively protects the PTFE-Eu^3+^ film from contacting with water molecules. The outstanding resistance is of significant importance since water or aqueous salt solution are the common service environments for rare earth materials such as *in vivo* and *in vitro* bioimaging applications^47^,^48^,^49^.

## Discussions

Superhydrophobic luminescent films with excellent water-resistance to water or aqueous salt solution were successfully prepared from rare earth-coordinated polymer nanoparticles and PTFE microparticles. The superhydophobic and self-cleaning surfaces of films were confirmed by the high water contact angle up to 161° and low sliding angle of about 6°. The films can emit strong red and green fluorescence with good color purity under UV excitation. More importantly, it is observed that the superhydrophobic rare earth polymer films can maintain high fluorescence stability when submersed in water or aqueous salt solution for two days. The results indicate that the superhydrophobic modification indeed effectively prevents water from touching the materials, and thus significantly enhances the resistance to water corrosion for the rare earth film. The combination of superhydrophobic and fluorescence properties as well as excellent fluorescence stability in aqueous environment is expect to greatly promote the applications of rare earth polymer composite materials in medical diagnosis, fluorescent probe, luminescent labels and many other fields.

## Methods

### Materials

Styrene (St) and acrylic acid (AA) were purchased from Aladdin Chemistry Co. and purified by vacuum-distillation prior to use. Europium nitrate and terbium nitrate were purchased from Shandong Qingda Fine Chemical Factory and dehydrated under vacuum at 140 °C prior to use. Potassium persulfate (KPS) was recrystallized from deionized water. Polytetrafluoroethylene (PTFE, 5 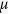
m, surface area of 7.5 m^2^ g^−1^), 1, 10-phenanthroline (Phen), acetylacetone, 1-hexadecanol (HD) and sodium dodeyl sulfate (SLS) were purchased from Aladdin Chemistry Co. and used as received.

### Instrumentation

Fourier transform infrared spectra (FTIR) were measured on an EQUINOX55 FT-IR spectrometer in the 400–4000 cm^−1^ region. Elemental analyses were performed on an Elementar Vario ELIII elemental analyzer. The contents of rare earth ions were measured by optimer 2000dv inductively coupled plasma (ICP). The sizes and distributions of particles were analyzed with dynamic light scatterings performed on a Malver Zetasizer Nano-ZS90 instrument at room temperature. Field-emission scanning electron micrographs (FE-SEM) were measured on a Nova NanoSEM 450. High-resolution transmission electron micrographs (HR-TEM) were measured on a JEM-2000EX instrument. The latexes were diluted to a ratio of 1:10000. Then the diluted suspension was coated on a copper grid for measurements. The chemical compositions of film surfaces were analyzed with energy-dispersive X-ray spectroscopy (EDX, FEI NOVA NanoSEM450). Thermal gravimetric analyses (TGA) were carried out on TA Q500 under nitrogen atmosphere in the temperature range from 50 to 700 °C at a heating rate of 10 °C min^−1^. Fluorescence emission spectra were measured using a PTI-700 fluorescence spectrophotometer with a scanning rate of 600 nm min^−1^ at room temperature. The microscopic fluorescence images were recorded with an OLYMPUS IX71 inverted fluorescence microscope equipped with a CCD camera. Water contact angles were measured using a contact angle goniometer (Dataphysics OCAH200) at room temperature. For each sample, two films were used for contact angle measurements. 4.5 μL Droplets were tested on 4–5 different positions on the surface, and the obtained values were averaged. The water sliding angles were recorded by the attached CCD camera on the goniometer. A high-speed digital camera attached to the contact angle goniometer was used to record the video and images of the water droplet (8 μL) rolling off the surface.

### Synthesis of core-shell polymer nanoparticles with carboxylic groups in shell layer

Synthesis of polystyrene seed latex: 0.25 g SLS, 0.835 g HD and 85 ml deionized water were charged into a 250 ml three-necked flask equipped with a mechanical stirrer, dropping funnels and a nitrogen inlet under vigorously stirring at 70 °C for 30 min. Then the solution was cooled to room temperature and 13 g St was added into the flask. After ultrasonic treatment for 3 min at 0 °C, the KPS aqueous solution (0.05 g of KPS in 5 ml water) was added, and the polymerization was allowed to proceed for 8 h at 70 °C to obtain polystyrene seed latex.

In the second step, the core-shell-structured nanoparticles with carboxylic groups in the shell layer was prepared according to the procedure: 20 ml deionized water, SDS (0.35 g), 5.2 g St and 1.8 g AA were introduced into a 50 ml three-neck round-bottom flask equipped with a mechanical stirrer, dropping funnels and a nitrogen inlet and stirred for 30 min. The obtained shell monomer pre-emulsion and 10 ml KPS aqueous solution (0.05 g of KPS in 15 mL water) were added continuously into the reactor containing polystyrene seed latex over a period of 3 h at 80 °C. After polymerization for 30 min, the solids were collected and washed with water and ethanol. Then the polymer particles were re-dispersed in ethanol to obtain a latex with a solid content of 3 wt%.

### Synthesis of rare earth polymer nanoparticles

Trivalent terbium ion (Tb^3+^) coordinated polymer complexes nanoparticles (Nano-Tb^3+^) were prepared according to the procedure: 10 g polymer nanoparticles latex (0.375 mmol carboxyl group), 0.129 g Tb(NO_3_)_3_ (0.375 mmol), 0.0675 g Phen (0.375 mmol) and 0.075 g acetylacetone (0.75 mmol) were mixed and stirred for 2 h at room temperature. After filtration, the solid was successively washed with ethanol and deionized water, and then the resultant particles were re-dispersed in ethanol to obtain Nano-Tb^3+^ latex.

The preparation of trivalent europium ion (Eu^3+^) coordinated polymer nanoparticles (Nano-Eu^3+^) is similar to that of (Nano-Tb^3+^).

### Preparation of PTFE/rare earth films

PTFE/Nano-Tb^3+^ composite films (PTFE- Tb^3+^) with different PTFE contents were prepared by adding PTFE in 10 mL Nano-Tb^3+^ colloidal solution. The mixture was dispersed with ultrasonic treatment for 15 min at room temperature. The resultant latex was casted on a clean slide glass, and allowed to dry at room temperature for 24 h. The PTFE/Nano-Eu^3+^ composite films were prepared with the similar procedure.

## Additional Information

**How to cite this article**: Wang, Z. *et al.* Fabrication of Superhydrophobic and Luminescent Rare Earth/Polymer complex Films. *Sci. Rep.* **6**, 24682; doi: 10.1038/srep24682 (2016).

## Supplementary Material

Supplementary Information

## References

[b1] Chen K. L., Zhou S. X., Yang S. & Wu L. M. Fabrication of All-Water-Based Self-Repairing Superhydrophobic Coatings Based on UV-Responsive Microcapsules. Adv. Funct. Mater. 25, 1035–1041 (2015).

[b2] Chen K. L., Zhou S. X. & Wu L. M. Facile Fabrication of Self-Repairing Superhydrophobic Coatings. Chem. Commun. 50, 11891–11894 (2014). 10.1039/c3cc49251f 25157381

[b3] Li Y., Duan G. T., Liu G. Q. & Cai W. P. Physical Processes-Aided Periodic Micro/Nanostructured Arrays by Colloidal Template Technique: Fabrication and Applications. Chem. Soc. Rev. 42, 3614–3627 (2013). 23403915 10.1039/c3cs35482b

[b4] Chu Z. L. & Seeger S. Superamphiphobic Surfaces. Chem. Soc. Rev. 43, 2784–2798 (2014). 24480921 10.1039/c3cs60415b

[b5] Sun T. L., Feng L., Gao X. F. & Jiang L. Bioinspired surfaces with special wettability. Acc. Chem. Rev. 38, 644–652 (2005). 10.1021/ar040224c 16104687

[b6] Tuteja A., Choi W. & Ma M. Designing superoleophobic surfaces. Science 318, 1618–1622 (2007). 18063796 10.1126/science.1148326

[b7] Li X. M., Reinhoudt D. & Crego-calama M. What do we need for a superhydrophobic surface? a review on the recent progress in the preparation of superhydrophobic surfaces. Chem. Soc. Rev. 36, 1350–1368 (2007). 17619692 10.1039/b602486f

[b8] Avinash M. B., Verheggen E., Schmuck C. & Govindaraju T. Self-cleaning functional molecular materials. Angew. Chem. Int. Ed. 51, 10324–10328 (2012). 10.1002/anie.201204608 22969032

[b9] Juan C., Tuberquia G. & Kane J. Surface initiation from adsorbed polymer clusters: a rapid route to superhydrophobic coatings. ACS Appl. Mater. Interfaces 5, 2593–2598 (2013). 23465650 10.1021/am303275f

[b10] Jonathan B. & Boreyko P. C. Delayed frost growth on jumping-drop superhydrophobic surfaces. ACS Nano 7, 1618–1627 (2013). 23286736 10.1021/nn3055048

[b11] Yang H. *et al.* Preparation of lotus-leaf-like antibacterial film based on mesoporous silica microcapsule-supported ag nanoparticles. RSC Adv. 4, 2793–2796 (2014).

[b12] Cong Y., Chen K. L., Zhou S. X. & Wu L. M. Synthesis of pH and UV Dual-Responsive Microcapsules with High Loading Capacity and Their Application in Self-Healing Hydrophobic Coatings. J. Mater. Chem. A 3, 19093–19099 (2015).

[b13] Sun Y. Y., Chen M., Zhou S. X., Hu J. & Wu L. M. Controllable Synthesis and Surface Wettability of Flower-Shaped Silver Nanocube-Organosilica Hybrid Colloidal Nanoparticles. ACS Nano 9, 12513–12520 (2015). 26564332 10.1021/acsnano.5b06051

[b14] Wen L., Tian Y. & Jiang L. Bioinspired super-wettability from fundamental research to practical applications. Angew. Chem. Int. Ed. 54, 3387–3399 (2015). 10.1002/anie.201409911 25614018

[b15] Wei Q. *et al.* Supramolecular polymers as surface coatings: rapid fabrication of healable superhydrophobic and slippery surfaces. Adv. Mater. 26, 7358–7364 (2014). 25236438 10.1002/adma.201401366

[b16] Nguyen J. G. & Cohen S. M. Moisture-resistant and superhydrophobic metal-organic frameworks obtained via postsynthetic modification. J. Am. Chem. Soc. 132, 4560–4561 (2010). 20232871 10.1021/ja100900c PMC2860283

[b17] Yohe S. T., Colson Y. L. & Grinstaff M. W. Superhydrophobic materials for tunable drug release: using displacement of air to control delivery rates. J. Am. Chem. Soc. 134, 2016–2019 (2012). 22279966 10.1021/ja211148a PMC3878812

[b18] Tuberquia J. C. *et al.* Surface-initiated polymerization of superhydrophobic polymethylene. J. Am. Chem. Soc. 132, 5725–5734 (2010). 20359210 10.1021/ja9086193

[b19] Zhu Q. *et al.* Robust superhydrophobic polyurethane sponge as a highly reusable oil-absorption material. J. Mater. Chem. A 1, 5386–5393 (2013).

[b20] Darmanin T. & Guittard F. recent advances in the potential applications of bioinspired superhydrophobic materials. J. Mater. Chem. A 2, 16319–16359 (2014).

[b21] Gai S., Li C., Yang P. & Lin J. Recent progress in rare earth micro/nanocrystals: soft chemical synthesis, luminescent properties, and biomedical applications. Chem. Rev. 114, 2343–2389 (2014). 24344724 10.1021/cr4001594

[b22] Pu F., Ju E., Ren J. & Qu X. Multiconfigurable logic gates based on fluorescence switching in adaptive coordination polymer nanoparticles. Adv. Mater. 26, 1111–1117 (2014). 24243760 10.1002/adma.201304109

[b23] Decadt R. *et al.* Synthesis, crystal structures, and luminescence properties of carboxylate based rare-earth coordination polymers. Inorg. Chem. 51, 11623–11634 (2012). 23078525 10.1021/ic301544q

[b24] Wang F. & Liu X. Recent advances in the chemistry of lanthanide-doped upconversion nanocrystals. Chem. Soc. Rev. 38, 976–989 (2009). 19421576 10.1039/b809132n

[b25] Haase M. & Schäfer H. Upconverting nanoparticles. Angew. Chem. Int. Ed. 50, 5808–5829 (2011). 10.1002/anie.201005159 21626614

[b26] Zhou J., Li Z. & Li F. Upconversion nanophosphors for small-animal imaging. Chem. Soc. Rev. 41, 1323–1349 (2012). 22008740 10.1039/c1cs15187h

[b27] Chen Z. *et al.* Versatile synthesis strategy for carboxylic acid-functionalized upconverting nanophosphors as biological labels. J. Am. Chem. Soc. 130, 3023–3029 (2008). 18278910 10.1021/ja076151k

[b28] Li K. & Liu B. Polymer-encapsulated organic nanoparticles for fluorescence and photoacoustic imaging. Chem. Soc. Rev. 43, 6570–6597 (2014). 24792930 10.1039/c4cs00014e

[b29] Li X. *et al.* Energy migration upconversion in manganese (II)-doped nanoparticles. Angew. Chem. Int. Ed. 54, 13312–13317 (2015). 10.1002/anie.201507176 26358961

[b30] Philippot C. *et al.* Doped silica nanoparticles containing two-photon luminescent Eu(III) complexes for the development of water stable bio-labels. J. Mater. Chem. 21, 18613–18622 (2011).

[b31] Chen M., Xie L., Li F. Y., Zhou S. X. & Wu L. M. Capillary-force-induced formation of luminescent polystyrene/(rare-earth-doped nanoparticle) hybrid hollow spheres. ACS Appl. Mater. Interfaces. 2, 2733–2737 (2010). 20828167 10.1021/am100726w

[b32] Bellusci A. *et al.* Synthesis and luminescent properties of novel lanthanide(iii) beta-diketone complexes with nitrogen P,P’-disubstituted aromatic ligands. Inorg. Chem. 44, 1818–1825 (2005). 15762708 10.1021/ic048951r

[b33] Scheirs J. Modern Fluoropolymers Wiley, New York (1997).

[b34] Bhushan B. & Jung Y. Natural and biomimetic artificial surfaces for superhydrophobicity, self-cleaning, low adhesion, and drag reduction. Prog. Mater. Sci. 56, 1–108 (2011).

[b35] Lee Y., Park S., Kim K. & Lee J. Fabrication of hierarchical structures on a polymer surface to mimic natural superhydrophobic surfaces. Adv. Mater. 19, 2330–2335 (2007).

[b36] Yildirim A., Budunoglu H., Daglar B., Deniz H. & Bayindir M. one-pot preparation of fluorinated mesoporous silica nanoparticles for liquid marble formation and superhydrophobic surfaces. ACS Appl. Mater. Interfaces 3, 1804–1808 (2011). 21574636 10.1021/am200359e

[b37] Petoud S., Cohen S. M., Bünzli J. C. G. & Raymond K. N. Stable lanthanide luminescence agents highly emissive in aqueous solution: multidentate 2-hydroxyisophthalamide complexes of SM^3+^, Eu^3+^, Tb^3+^, Dy^3+^. J. Am. Chem. Soc. 125, 13324–13325 (2003). 14583005 10.1021/ja0379363

[b38] Liu D. & Wang Z. Novel polyaryletherketones bearing pendant carboxyl groups and their rare earth complexes, Part I: Synthesis and characterization. Polymer 49, 4960–4967 (2008).

[b39] Wang L. H., Wang W., Zhang W. G., Kang E. T. & Huang W. Synthesis and luminescence properties of novel Eu-containing copolymers consisting of Eu(III)-acrylate-beta-diketonate complex monomers and methyl methacrylate. Chem. Mater. 12, 2212–2218 (2000).

[b40] Yan B., Zhang H. J., Wang S. B. & Ni J. Z. luminescence properties of the ternary rare earth complexes with beta-diketones and 1,10-phenanthroline incorporated in silica matrix by a sol-gel method. Mater. Chem. Phys. 51, 92–96 (1997).

[b41] Xiao Z., Tan S., Zou Y., Zhao B. & Wang X. Study on the effec of α-allyl of luminescence properties of rare earth complex with acetylacetone. Spectrosc. Spect. Anal. 24, 18–20 (2004). 15768966

[b42] Liu Y., Xin J. & Choi C. Cotton fabrics with single-faced superhydrophobicity. Langmuir 28, 17426–17434 (2012). 23186211 10.1021/la303714h

[b43] Ming W., Lou X., Grampel R. D., Dongen J. L. J. & Linde R. Partial fluorination of hydroxyl end-capped oligoesters revealed by MALDI−TOF mass spectrometry. Macromolecules 34, 2389–2393 (2001).

[b44] Sas L., Gorga R., Joines J. & Thoney K. Literature review on superhydrophobic self-cleaning surfaces produced by electrospinning. J. Polym. Sci. B: polym. Phys. 50, 824–845 (2012).

[b45] Sparks J., Hoff E., Xiong L., Goetz J. & Patton D. Superhydrophobic hybrid inorganic-organic thiol-ene surfaces fabricated via spray-deposition and photopolymerization. ACS Appl. Mater. Interfaces 5, 1811–1817 (2013). 23410965 10.1021/am303165e

[b46] Zhang Y. *et al.* Solvothermal synthesis of nanoporous polymer chalk for painting superhydrophobic surfaces. Langmuir 27, 12585–12590 (2011). 21875116 10.1021/la2018264

[b47] Xia A. *et al.* Enhanced dual contrast agent, Co^2+^-doped NaYF4:Yb^3+^, Tm^3+^ nanorods, for near infrared-to-near infrared upconversion luminescence and magnetic resonance imaging. Biomaterials 35, 9167–9176 (2014). 25108318 10.1016/j.biomaterials.2014.07.031

[b48] Nyk M., Kumar R., Ohulchanskyy T. Y., Bergey E. J. & Prasad P. N. High contrast *in vitro* and *in vivo* photoluminescence bioimaging using near infrared to near infrared up-conversion in tm3^+^ and yb3^+^ doped fluoride nanophosphors. Nano Lett. 8, 3834–3838 (2008). 18928324 10.1021/nl802223f PMC3523349

[b49] Li R. *et al.* Enhancing the imaging and biosafety of upconversion nanoparticles through phosphonate coating. ACS Nano 9, 3293–3306 (2015). 25727446 10.1021/acsnano.5b00439 PMC4415359

