# Supplementary material for: Fabrication of Superhydrophobic and Luminescent Rare Earth/Polymer complex Films
Source: Sci Rep. 2016 Apr 18;6:24682. doi: 10.1038/srep24682 (PMC5263856; doi:10.1038/srep24682)
Supplement: Supplementary Information [file srep24682-s1.pdf]

Supporting Information

Fabrication of Superhydrophobic and Luminescent Rare Earth/Polymer Complex Films

Zefeng Wang, Weiwei Ye, Xinran Luo & Zhonggang Wang*

Department of Polymer Science and Materials, School of Chemical Engineering, Dalian University of Technology, Dalian 116024, China

Email: zgwang@dlut.edu.cn

Contents

Table S1 Elemental compositions of rare earth-coordinated polymer nanoparticles.

Figure S1. FTIR spectra of (a) Acac, (b) Phen, (c) PS-*co*-PAA nanoparticle and (d) Nano-Eu^{3+} nanoparticle.

Figure S2. DLS curves for core polymer particles, carboxyl-containing core-shell polymer particles, and rare earth-coordinated polymer particles.

Figure S3. TGA curves of (a) Nano-Eu^{3+} and PTFE-Eu^{3+} films.

Figure S4. Images of PTFE-Eu^{3+} and Nano-Eu^{3+} films just pulled out of water.

Figure S5. Fluorescence emission spectra of Nano-Eu^{3+} film after submersed in water for different time.

Figure S6. Fluorescence emission spectra of PTFE-Eu^{3+} film after submersed in 20% aqueous NaCl solution for different time.

Table S1. **Elemental compositions of rare earth-coordinated polymer nanoparticles.**

Sample	Element	Found (%)	Calc. (%)
Nano-Eu^{3+}	C	65.22	64.07
	H	5.62	5.47
	N	4.84	4.61
	Eu	11.06	10.02
Nano-Tb^{3+}	C	66.17	63.78
	H	5.76	5.45
	N	4.29	4.59
	Tb	10.88	10.43

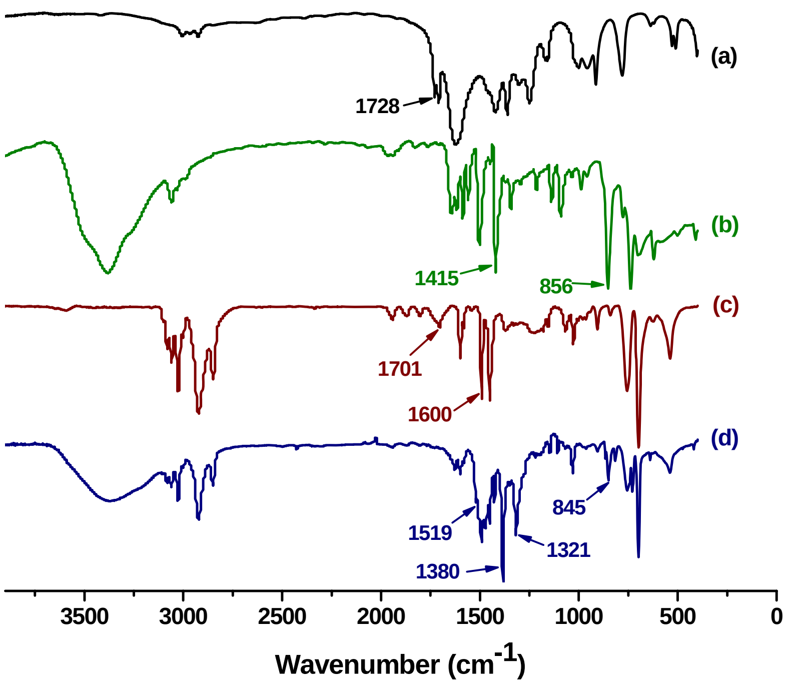

Figure S1. FTIR spectra of (a) Acac, (b) Phen, (c) PS-*co*-PAA nanoparticle and (d) Nano-Eu^{3+} nanoparticle.

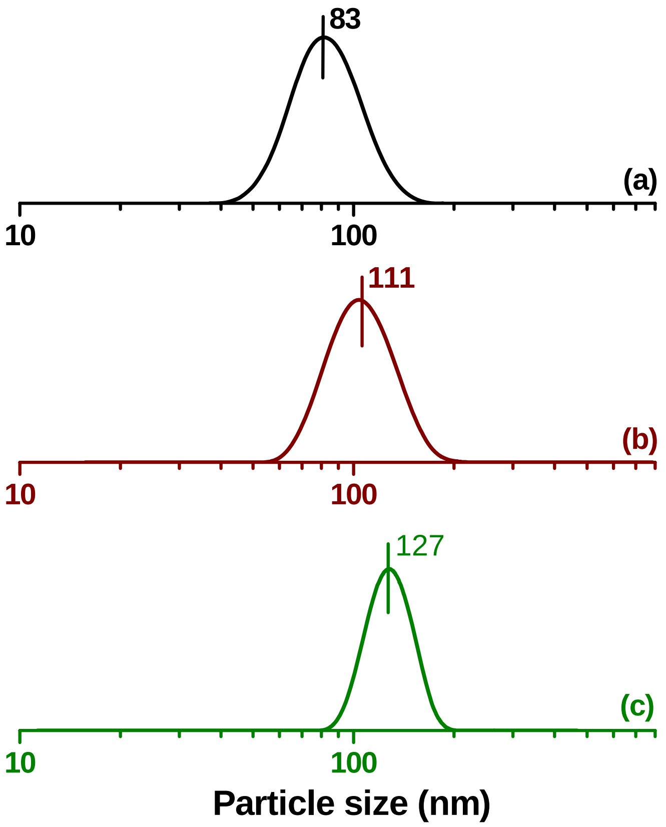

Figure S2. DLS curves for core polymer particles (a), carboxyl-containing core-shell polymer particles (b), and rare earth-coordinated polymer particles (c).

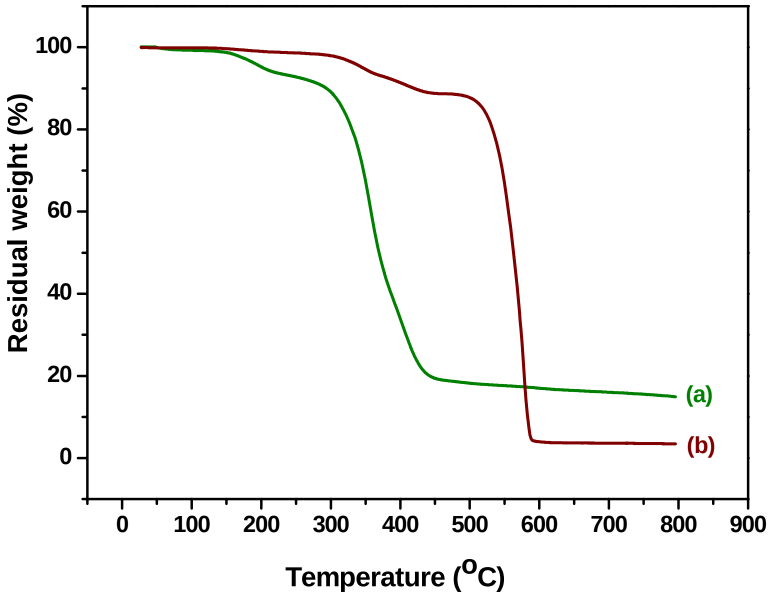

Figure S3. TGA curves of (a) Nano-Eu^{3+} and PTFE-Eu^{3+} films.

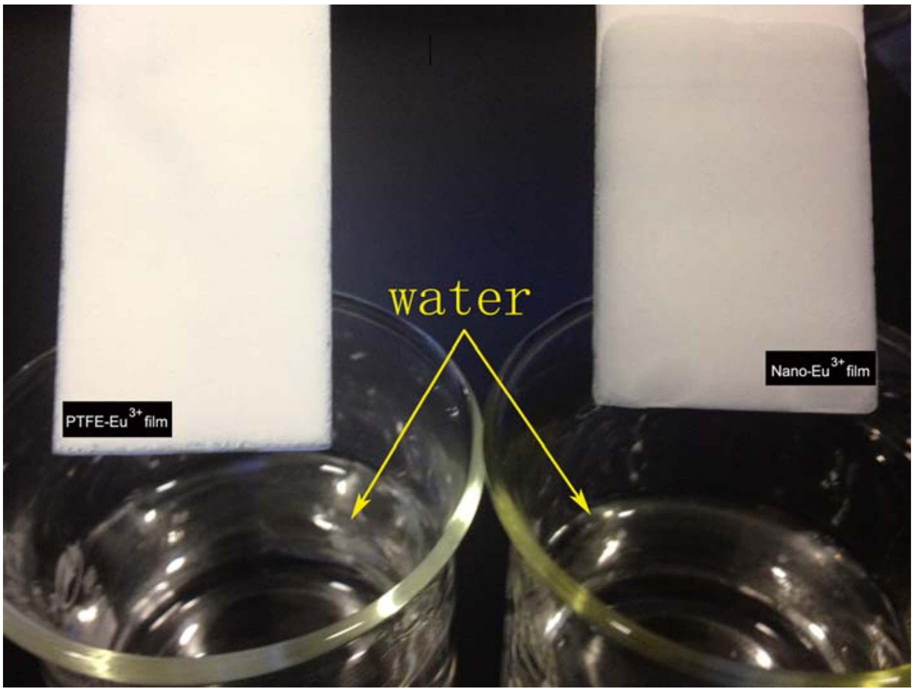

Figure S4. Images of PTFE-Eu^{3+} and Nano-Eu^{3+} films just pulled out of water.

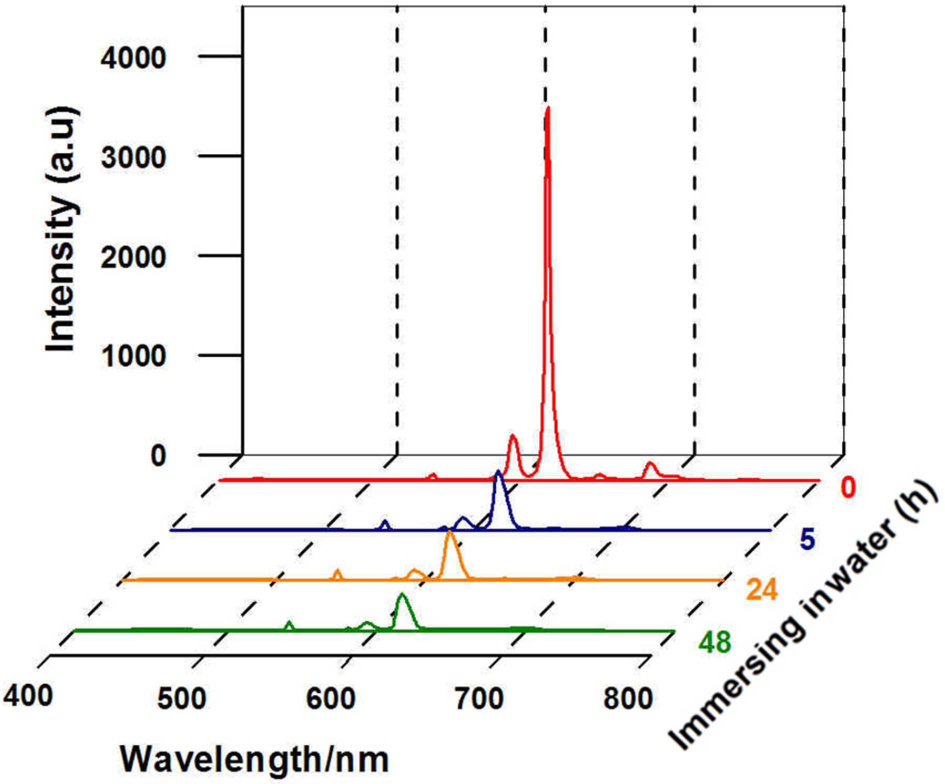

Figure S5. Fluorescence emission spectra of Nano-Eu^{3+} film after submersed in water for different time.

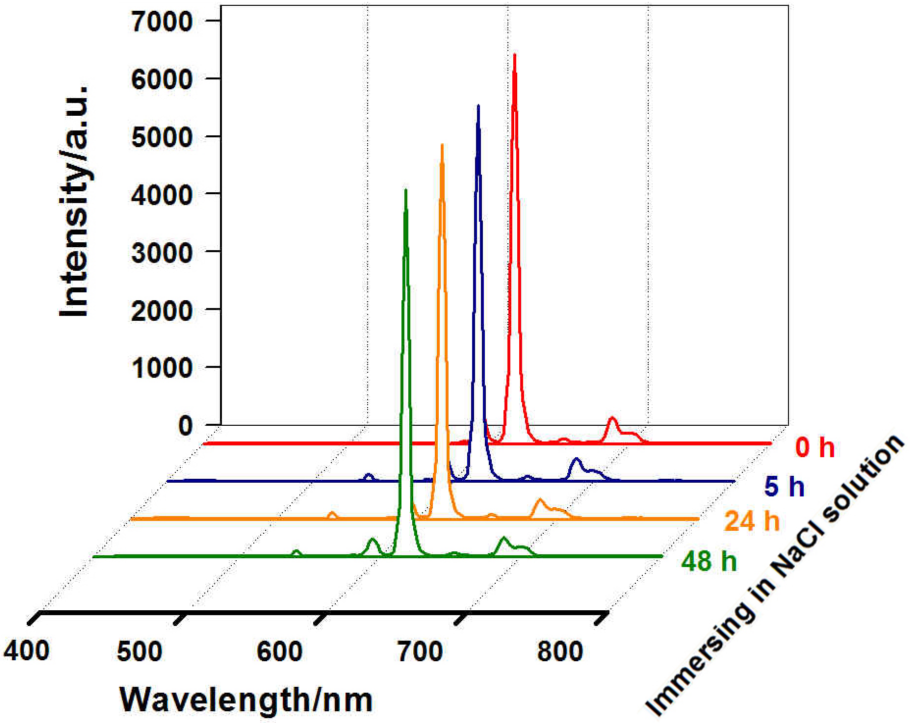

Figure S6. Fluorescence emission spectra of PTFE-Eu^{3+} film after submersed in 20% aqueous NaCl solution for different time.
